# A Comprehensive Investigation on the Fire Hazards and Environmental Risks in a Commercial Complex Based on Fault Tree Analysis and the Analytic Hierarchy Process

**DOI:** 10.3390/ijerph17197347

**Published:** 2020-10-08

**Authors:** Yongyu Wang, Xiaoyang Ni, Jie Wang, Ziyi Hu, Kaihua Lu

**Affiliations:** 1 Faculty of Engineering, China University of Geosciences, Wuhan 430074, China; yywang@cug.edu.cn (Y.W.); xy_ni@163.com (X.N.); huzy1995@163.com (Z.H.); 2 School of Resource and Environmental Engineering, Wuhan University of Science and Technology, Wuhan 430081, China

**Keywords:** fire risk assessment, commercial complex, fault tree analysis, analytic hierarchy process

## Abstract

This paper focuses on the fire risk assessment for commercial complex, as the variety of fire accidental triggers inside could be a big threat to the public fire safety, leading to catastrophic loss in human lives and properties. Both the qualitative and quantitative analysis were imposed on a typical large commercial complex to recognize the potential fire-causative factors in this paper. Applying the fault tree analysis, the basic events leading to fire are acquired, and they are then further reclassified based on the analytic hierarchy process. Taking the damage of the accident as the target layer and the fire-causative factors, the equipment operation factors and firefighting factors as the criterion layer, the assessment index is well established. The risk of each factor is quantitatively evaluated, and the effect of each factor on the target layer is analyzed. The result of the fault tree analysis and analytic hierarchy process shows good consistency, in which human behavior is the main factor leading to the fire occurrence, followed by the combustible material, the rescue speed and the staff assignment factors. The results are beneficial for general decisions and measures in public fire safety management.

## 1. Introduction

The rapid development of the Chinese economy has greatly enriched people’s daily life, with a large number of commercial complexes in the city built up to meet the daily needs of people’s consumption and entertainment. These commercial complexes with a large building space, a variety of complex functions, intensive personnel activities as well as other characteristics, contain a variety of accidental triggers that could easily lead to diverse types of accidents, with fire accidents occupying a significant proportion of among them. 

In recent years, fire accidents of various commercial buildings and complexes have been emerging one after another, resulting in a large amount of economic losses, posing a serious threat to the public fire safety of the masses, and drawing wide attention from society. In addition, in line with fire occurrence, large amount of smoke and toxic gases, including CO, CO_2_, HCN, SO_2_, HCl and NO_x_ etc., could be also harmful for the public health (e.g., the breathing system, visual system and nervous system for human beings) in the building, which is also a main reason for death during a fire accident [1]. For example, in March 2018, a fire accident occurred in a 4-floor commercial complex containing a shopping center, restaurants, retail stores and cinema in Western Siberia of Russia, causing 64 deaths and 52 injuries [2], whereas in August 2020, even though nobody was killed, the commercial building fire in Qingyuan, Guangdong province of China, led to a catastrophic fire that spread on the building façade, causing massive property losses [3]. Unlike individually functioning retail shops, the reason to fire accidents in commercial complexes could be much more piecemeal, for example, the improper use of fire by human activity or unrestricted combustibles (e.g., smoking, welding working, kitchen working, and combustible material) or the failure in fire safety and rescue systems (e.g., the error in fire detection, fire alarms and fire equipment or deficit in rescue team). Thus, the consequential fire damage degree could be very different. However, the problem is that the fire risk in commercial complex areas has not been well established, and the assessment result remains unclear, making the public fire safety management unsound. It is worthwhile to carry out research on fire hazard analysis and evaluation of such commercial complexes, as it could be helpful for the general decisions and measures regarding public fire safety management.

At present, scholars have carried out some fire risk assessment method investigations for different buildings [4,5,6,7,8,9,10,11]. Generally, the risk assessments can be divided into two categories, i.e., the qualitative methods and the quantitative methods. In the series of qualitative ones, the most representative is fault tree analysis (FTA) [12,13,14], which has been widely used in risk assessment [15,16,17]. The core of FTA is to list the causative factors layer by layer just like a tree, including the top event (which is also the so-called “accident”), the intermediate events, as well as the basic factors in which the events are linked to each other through logical gates. Hence, we could search the minimal path set (the shortest set through which the top event would not be taken place) to avoid the occurrence of the top event by Boolean operation (a kind of logical operation for the set in mathematics). However, the shortcoming of FTA is that the specific importance level that each basic factor occupies remains unknown, as while they are all listed in the lowest layer belonging to different intermediate events, there is no way to compare them. On the other hand, the logical operation would become more extensive as more and more factors are taken into consideration in FTA. 

On the contrary, for the quantitative methods, the relative importance weight of factors could be acquired much easier. For instance, Saaty [18] identified a quantitative way to make the comparison among accidental factors much easier and more intuitive, namely, the analytic hierarchy process (AHP), which has been applied in a number of building fire risk assessments. For example, Tang [19] conducted an assessment of the fire risk in village in Shenzhen based on the AHP. Tian [20] developed the AHP algorithm based on the fire risk assessment of a shopping mall. Xu [21] carried out fire risk identification and AHP assessment in historic buildings. More recently, the AHP was also introduced to the natural fire disaster in Inner Mongolia [22]. Nevertheless, the limitation of the AHP is that it is only usable for a three-layer-system, i.e., the target layer, the criteria layer and the index layer, making it significantly simpler than FTA. The reduction in logical layers may make the risk assessment somewhat less accurate. Current research rarely use both qualitative and quantitative methods in risk assessment; thus, the risk assessment result may somewhat not reasonable. 

Therefore, we suggest incorporating FTA and the AHP together to increase the accuracy of the risk assessment. In this paper, taking a typical large-scale commercial complex as the research background, the importance order of each fire incentive is qualitatively obtained based on the FTA method; then, the basic causative factors identified by FTA are reclassified into different layers belonging to different criteria layers, with the specific weight of each factor index acquired quantitatively by the AHP. The combination of FTA and the AHP could be further applied in similar cases, providing a significance reference and theoretical basis, which is beneficial for general decisions and measures regarding public fire safety management in commercial complexes.

## 2. A Brief Overview of the Study Area

The following fire risk assessment is based on a large commercial complex named Wushangzhongyuan Square in Wuhan City, Hubei Province of China. This is one of the largest commercial complex serving shopping, catering, entertainment, sporting, leisure, education and exhibition complexes in the Qingshan District in Wuhan City. The selection of this commercial complex was due to the fact that it was listed in the high-risk fire unit by the local fire brigade in May 2020 [23]. The commercial complex consists of two buildings with a total business area of 270,000 m^2^. The over ground part is six floors of 180,000 m^2^, containing retail stores of luxury items, men’s wear, and women’s wear on the first to third floor; baby’s clothing stores, early education and a playground for children on the fourth floor; catering restaurants, bars, KTV and a cinema on the fifth floor; and a leisure and sporting space on the sixth floor. Moreover, the underground part consists of two floors of 90,000 m^2^, with a supermarket, some catering restaurants and a parking lot. The operation information of the commercial complex were well investigated, and the basic events leading to fire were obtained and classified.

## 3. Fault Tree Analysis (FTA)

### 3.1. Establishment of the Fault Tree

**Figure 1:**
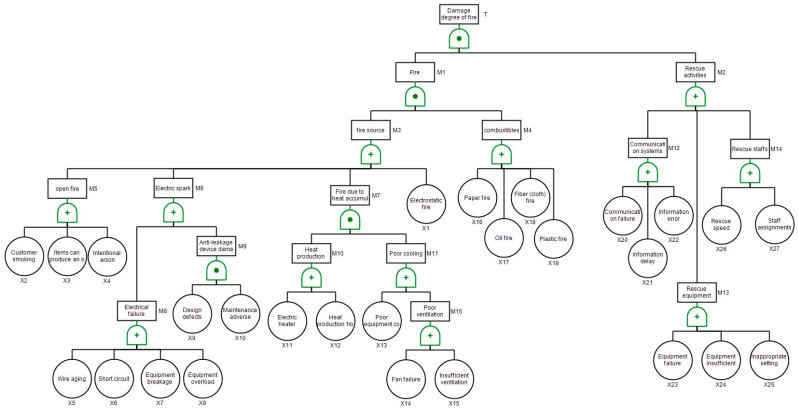
The establishment of the fault tree. T: In FTA, firstly the “damage degree of the accident (fire)” is set as the top event, denoted as T.

In FTA, firstly the “damage degree of the accident (fire)” is set as the top event, denoted as T. Secondly, since the damage degree of the fire should be relevant to fire and rescue activities, “fire” and “rescue activities” are considered as a second-level intermediate events, denoted M1 and M2, respectively. Moreover, the intermediate events “fire” and “rescue activities” will be further expanded to form inferior-level intermediate events, for example, the intermediate events of “fire source” and “combustibles” belong to the intermediate events of “fire”, whereas the intermediate events of “communication systems”, “rescue equipment” as well as “rescue staffs” are in the next level of “rescue activities”. All the intermediate events marked by M1–M15 are listed in Table 1, and the basic factors (X1–X27) are classified below the level of the intermediate events, as presented in Table 2. Finally, to complete the fault tree, the relationship between the events is logically analyzed, and the logical gate is employed in linking the top event, intermediate events and the basic events, as depicted in Figure 1.

### 3.2. The Qualitative Analysis on the Importance of Each Basic Event

Here, we can see that the fire-causative factors and the connections are well presented in the established fault tree. In order to gain a better understanding of the importance of each event, the minimal path set method (the shortest set through which the top event would not be taken place) executed by the Boolean operation (which is a logical operation for the set in mathematics) is applied in simplification in FTA. The minimal path set includes the following six sets:

(1) $$\left\{ \begin{array}{l} P1=\left\{X1,X2,X3,X4,X5,X6,X7,X8,X9,X11,X12\right\};\\ P2=\left\{X1,X2,X3,X4,X5,X6,X7,X8,X9,X13,X14,X15\right\};\\ P3=\left\{X1,X2,X3,X4,X5,X6,X7,X8,X10,X11,X12\right\};\\ P4=\left\{X1,X2,X3,X4,X5,X6,X7,X8,X10,X13,X14,X15\right\};\\ P5=\left\{X16,X17,X18,X19\right\};\\ P6=\left\{X20,X21,X22,X23,X24,X25,X26,X27\right\} \end{array}\right.$$

We note that the minimal path set P5 contains only four elements, which is the smallest number of elements among the six sets; therefore, qualitatively, all the elements in P5 (X16–X19) are of the highest structural significance, because once a single event of X16–X19 occurs, logically, the top event would take place, subsequently causing fire damage. Then, the structural importance of the elements in the minimal path set P6 (X20–X27) is in the second place. Using the same judge criterion, all the other elements are divided into another four series: (1) the basic factors X11 and X12 form the third structural importance level; (2) X1–X8 should be positioned in the fourth structural importance level; (3) The elements X9 and X10 are placed in the fifth structural importance level, as they are present in the minimal path sets P1 and P3 which contain the secondary numbers of the events; and (4) the elements X13–X15 are of the lowest structural importance among the 27 basic factors as they exist in the longest two minimal path sets, P2 and P4. Eventually, the order of structural importance of each basic event is as follows:

(2) $$\begin{array}{l} \;I\left(X19\right)=I(X18)=I(X17)=I(X16)\\ >I(X27)=I(X26)=I(X25)=I(X24)=I(X23)=I(X22)=I(X21)=I(X20)\\ >I(X8)=I(X7)=I(X6)=I(X5)=I(X4)=I(X3)=I(X2)=I(X1)\\ >I(X12)=I(X11)\\ >I(X10)=I(X9)\\ >I(X15)=I(X14)=I(X13) \end{array}$$

Hence, as discussed above, the most essential fire-causative basic factors are: “plastic fire”, “fiber (clothing fabric) fire”, “oil fire” and “paper fire”, respectively, which all belong to the combustible materials, indicating that more attention should be paid to the utilization of such materials in the case of fire prevention and public safety management in the commercial complex. However, as mentioned above, the FTA result could only provide such structural importance of each element—an understanding of the actual importance of the level is still not available. Hereby, the quantitative AHP method is presented in the next section.

## 4. Analytic Hierarchy Process (AHP)

### 4.1. The Judgment Matrix Based on the Comparison between the Fire-Causative Factors

Here, the above fire risk assessment of the commercial complex will be presented by the AHP to quantitatively verify the results from FTA. Proposed by the American operations researcher Saaty [18] in the 1970s, the AHP allows for the complex thinking process to be assessed in mathematical ways, greatly simplifying the analysis. 

To perform the AHP, firstly, the basic factors which can increase fire damage based on the FTA result can be rearranged and set up in a hierarchical structure model, as listed in Table 3. Here, layer A is the overall target layer of the damage degree of the accident (fire). Layer B is the criteria layer, including the “fire-causative factors” (B1), the “equipment operation factors” (B2) and the “firefighting factors” (B3). Then, layer C is the index layer, corresponding to all kinds of basic factors; for instance, basic factors such as “customer smoking” (X2) and “intentional arson” (X4) are categorized to the index of “human behavior” (C11).

We can see now that the relative importance of the three factors in the criteria layer is required to be pairwise compared. A judgment matrix [A] is introduced in this place:

(3) $$A=\left[\begin{array}{ccc} A_{11} & A_{12} & A_{13}\\ A_{21} & A_{22} & A_{23}\\ A_{31} & A_{32} & A_{33} \end{array}\right]$$

where the relative importance of factor Bi to Bj is denoted as Aij (i, j = 1, 2, …n). A quantitative method for a comparison between factors using the judgment scale is displayed in Table 4. 

The judgment matrix for the criteria layer for this case is considered as follows: being aware of the fact that the “fire-causative factors” (B1) are more essential than “firefighting factors” (B2), because fire-causative factors could lead to a fire directly, while the firefighting should start directly after the fire. The “equipment operation factors” (B3) plays a relative less important role in a fire accident, since the failure in equipment operation could start the electrical fire, but such electrical fires do not always happen.

(4) $$A=\left[\begin{array}{ccc} 1 & 4 & 2\\ 1/4 & 1 & 1/3\\ 1/2 & 3 & 1 \end{array}\right]$$

Moreover, the judgment matrix (B1–B3) can be built up using the same comparison method on the indexes of C11–C31, C12–C52 and C13–C43. We suggest the C11 (human behavior) is the most significant in the elements of C11–C31 because this part is less controllable. as smoking and intentional arson could not be completely forbidden in practice. Thus, in the indexes of C12–C52, we consider both the equipment load (C12) and equipment condition (C22) as the most important because they are more essential for electric fire factors than the others. Besides, in the indexes of C13–C43, the rescue speed (C13) and staff assignment (C23) determine the rescue activity directly, whereas although the fire equipment (C33) and fire alarm (C43) could be also helpful for the rescue condition, they are not always the determining factors.

(5) $$B_{1}=\left[\begin{array}{ccc} 1 & 3 & 7\\ 1/3 & 1 & 5\\ 1/7 & 1/5 & 1 \end{array}\right]$$

(6) $$B_{2}=\left[\begin{array}{ccccc} 1 & 1 & 2 & 3 & 4\\ 1 & 1 & 3 & 3 & 4\\ 1/2 & 1/3 & 1 & 1 & 3\\ 1/3 & 1/3 & 1 & 1 & 3\\ 1/4 & 1/4 & 1/3 & 1/3 & 1 \end{array}\right]$$

(7) $$B_{3}=\left[\begin{array}{cccc} 1 & 1 & 3 & 3\\ 1 & 1 & 3 & 3\\ 1/3 & 1/3 & 1 & 4\\ 1/3 & 1/3 & 1/4 & 1 \end{array}\right]$$

### 4.2. Calculation of the Relative Weight for Each Factor

The most important issue in the AHP is to confirm the relative weight of each factor from the proposed judgment matrix. Normally, we can calculate the weight eigenvector for the matrix through the following “n-th root” method [18]:

(1) Multiply the importance degree of each line along the matrix, which gives
  (8) $$W_{i}=\prod_{i=1}^{n}a_{ij}\;\left(i=1,2\ldots n\right)$$
(2) Calculated n-th root of the above result:
  (9) $${\bar{W}}_{i}=\sqrt[{n}]{W_{i}}\;\left(i=1,2\ldots n\right)$$
(3) Finally, each weight of the factor should be normalized by

(10) $$w_{i}=\frac{{\bar{W}}_{i}}{\sum_{j=1}^{n}{\bar{W}}_{i}}\;\left(i=1,2\ldots n\right)$$

which forms the weight eigenvector as $W={\left(w_{1},w_{2},w_{3}\ldots w_{n}\right)}^{T}$.

The last step before we finish the AHP for the fire risk assessment of the commercial complex is to determine whether the judgment matrix and the weight eigenvector are reasonable, noting that the comparison between the factors should be carefully input. Satty [18] suggested the consistency ratio (CR) should be taken into consideration at this point, where the CR is the ratio of the consistency index of the judgment matrix (CI) to the random consistency index (RI), i.e., CR = CI / RI. When the CR < 0.1, we can see that the judgment matrix shows good consistency for the various fire-causative factors. However, on the contrary, if the CR ≥ 0.1, the consistency of the judgment matrix is not good enough, indicating that the judgment matrix should be rebuilt. The random consistency index (RI) is provided in Table 5, while the consistency index of the judgment matrix (CI) can be obtained through the following expressions:

(11) $$CI=\frac{\lambda_{\max}-n}{n-1}$$

where n is the order of the judgment matrix, and λ_max_ is the maximum eigenvalue of the judgment matrix expressed by

(12) $$\lambda_{\max}=\frac{1}{n}\sum_{i=1}^{n}\frac{{\left(Aw\right)}_{i}}{w_{i}}\;\left(i=1,2,3\ldots n\right)$$

Taking judgment matrix A for example, the weight eigenvector of the criteria layer to the target layer is calculated to be $W_{A}={\left(0.558,\;0.112,\;0.320\right)}^{T}$. Thus, the λ_max_ of judgment matrix A is 3.019; therefore, judgment matrix A can pass the consistency check, as the consistency ratio (CR) is found to be 0.016, which is much smaller than 0.1. Repeating the same method above, the weight of the factors in the index layer to the criteria layer are obtained, as shown in Table 6, Table 7 and Table 8.

The weight of the factors in the index layer in relation to the target layer (WA, Cij) (i, j = 1, 2…n) are then as listed in Table 9, multiplying the weight of the criteria layer to the target layer (WA–Bi) (i, j = 1, 2…n), and the weight of the index layer to the criteria layer (WBi–Cij) (i, j = 1, 2…n), respectively. The results show that “human behavior” is the most significant factor in the case of a fire, while the “combustibles” remain at second place. From Table 3, it is clear that we should pay more attention to the following six factors: “customer smoking” X2, “intentional arson” X4, “paper fire” X16, “oil fire” X17, “fiber (cloth) fire” X18 and “plastic fire” X19. On the other hand, “rescue speed” and “staff assignment” are in the third place, corresponding to the basic factors X26 and X27 in FTA.

## 5. Discussion

Both FTA and the AHP indicate that to limit the amount of combustibles is essential, because both the structural importance and the weight in relation to target layer on which the factor stands are in prior placements according to the assessment result. Furthermore, the results from FTA and the AHP guide us to focus more on the fire equipment, the rescue activity and human behaviors. We believe the public fire safety management of commercial complexes has some improvement space in the following aspects:

(1) The effective supervision of human behavior should be strengthened. Human behavior includes two aspects: one is customer smoking, and the other is kitchen fire. In response to such problems, warning signs can be set up in the obvious places in the building to remind customers of the ban on smoking. Furthermore, the fire hazard in the catering in the upper floors should be also supervised.

(2) The fire load (heat release rate) should be under control. We should also focus on the fire hazards in clothing stores, underground supermarkets, warehouses, children’s playgrounds and other facilities to prevent fire from occurring. Fire extinguishers need to be properly distributed, and regular maintenance of the fire extinguishing equipment, sprinkler systems, smoke control systems and fire detection systems should be carried out to ensure that they are available in the case of a fire.

(3) Fire safety training of staff who are working in the commercial complex should be enhanced, including the basic skills of fire prevention and evacuation. Fire safety responsibility should be improved to the utmost in order to reduce the probability of fire occurrence.

In summary, from the results of the FTA and AHP combinations, the fire hazards in the commercial complex are identified more clearly. We believe the combination of FTA and the AHP could provide a good example for similar commercial complex fire risk assessments, and the implementation of such would be of significant supplementary value to the current research.

## 6. Conclusions

This paper carries out a fire risk assessment on a commercial complex based on the FTA and AHP methods. The main factors that can increase accident damages have been investigated and classified into different groups by studying the internal logical connections between them, while the importance degrees of the factors are presented in both qualitative and quantitative ways. The research shows that FTA can propose a qualitative judgment on the important degree of the basic factors, whereas the fire risk assessment index system established by the AHP provides an easy and quantitative supplement to the result of FTA, making the results more reasonable. The combination of FTA and the AHP shows good agreement, indicating that human behavior, the combustibles, the rescue speed and staff assignment are the highest significant elements, while also providing general decisions and measures in public fire safety management in this kind of commercial complex.

## Figures and Tables

**Table 1:** Intermediate events.

Intermediate Events	Mark	Intermediate Events	Mark	Intermediate Events	Mark
Fire	M1	Electric spark	M6	Poor cooling	M11
Rescue activities	M2	Fire due to heat accumulation	M7	Communication systems	M12
Fire source	M3	Electrical failure	M8	Rescue equipment	M13
Combustibles	M4	Anti-leakage device damage	M9	Rescue staffs	M14
Open fire	M5	Heat production	M10	Poor ventilation	M15

**Table 2:** Basic factors.

Basic Factors.	Mark	Basic Factors	Mark	Basic Factors	Mark
Electrostatic fire	X1	Maintenance adverse	X10	Plastic fire	X19
Customer smoking	X2	Electric heater	X11	Communication failure	X20
Items can produce an open fire	X3	Heat production from operating equipment	X12	Information delay	X21
Intentional arson	X4	Poor equipment cooling	X13	Information error	X22
Wire aging	X5	Fan failure	X14	Equipment failure	X23
Short circuit	X6	Insufficient ventilation	X15	Equipment insufficiency	X24
Equipment breakage	X7	Paper fire	X16	Inappropriate setting	X25
Equipment overload	X8	Oil fire	X17	Rescue speed	X26
Design defects	X9	Fiber (cloth) fire	X18	Staff assignments	X27

**Table 3:** The rearranged hierarchical structure with various basic factors.

Target Layer (A)	Criteria Layer (B)	Index Layer (C)	Basic Factors
Damage degree of the accident (fire) A	Fire-causative factors B1	Human behavior C11	Customer smoking X2, Intentional arson X4
		Combustibles C21	Paper fire X16, Oil fire X17, Fiber (cloth) fire X18, Plastic fire X19
		Open fire C31	Electrostatic fire X1, Items can produce an open fire X3
	Equipment operation factors B2	Equipment load C12	Equipment overload X8
		Equipment condition C22	Wire aging X5, Short circuit X6, Equipment breakage X7, Design defects X9
		Heat C32	Electric heater X11, Heat production from operating equipment X12, Poor Equipment cooling X13
		Ventilation C42	Fan failure X14, Insufficient ventilation X15
		Maintenance C52	Maintenance adverse X10
	Firefighting factors B3	Rescue speed C13	Rescue speed X26
		Staff assignment C23	Staff assignment X27
		Fire equipment C33	Equipment failure X23, Equipment insufficiency X24, Inappropriate setting X25
		Fire alarm C43	Communication failure X20, Information delay X21, Information error X22

**Table 4:** The rearranged hierarchical structure with various basic factors.

Judgment Scale	Meaning
1	Compare factor i with factor j, and it is of the same importance; thus Aij = 1.
3	Compare factor i with factor j, and i is slightly more important than j; thus Aij = 3.
5	Compare factor i with factor j, and i is clearly more important than j; thus Aij = 5.
7	Compare factor i with factor j, and i is strongly more important than j; thus Aij = 7.
9	Compare factor i with factor j, and i is extremely more important than j; thus Aij = 9.
2,4,6,8	The intermediate values of the two adjacent judgments mentioned above.
Note	The judgment scale follows the reciprocal relationship of Aji = 1 / Aij. When i = j, Aij = 1 (i, j = 1, 2…n).

**Table 5:** Random consistency index (RI).

n	1	2	3	4	5	6	7	8	9	10	11
RI	0	0	0.58	0.90	1.12	1.24	1.32	1.41	1.45	1.49	1.51

**Table 6:** Weight calculation results of “fire-causative factors”.

Judgment Scales	C11 Human Behavior	C21 Combustibles	C31 Open Fire	Weight
C11 human behavior	1	3	7	0.649
C21 combustibles	1/3	1	5	0.279
C31 open fire	1/7	1/5	1	0.072

CR = 0.056 < 0.1, consistency check passed. CR: consistency ratio.

**Table 7:** Weight calculation results of “equipment operation factors”.

Judgment Scales	C12 Equipment Load	C22 Equipment Condition	C32 Heat	C42 Ventilation	C52 Maintenance	Weight
C12 equipment load	1	1	2	3	4	0.316
C22 equipment condition	1	1	3	3	4	0.342
C32 heat	1/2	1/3	1	1	3	0.146
C42 ventilation	1/3	1/3	1	1	3	0.134
C52 maintenance	1/4	1/4	1/3	1/3	1	0.088

CR = 0.020 < 0.1, consistency check passed.

**Table 8:** Weight calculation results of “firefighting factors”.

Judgment Scales	C13 Rescue Speed	C23 Staff Assignment	C33 Fire Equipment	C43 Fire Alarm	Weight
C13 rescue speed	1	1	3	3	0.369
C23 staff assignment	1	1	3	3	0.369
C33 fire equipment	1/3	1/3	1	4	0.174
C43 fire alarm	1/3	1/3	1/4	1	0.174

CR = 0.090 < 0.1, consistency check passed.

**Table 9:** Weight of each factor in the index layer.

Target Layer	Criteria Layer	Weight to Target Layer (WA, Bi) (i, j = 1, 2…n)	Index Layer	Weight to Criteria Layer (WBi, Cij) (i, j = 1, 2…n)	Weight to Target Layer (WA, Cij = WA, Bi × WBi, Cij) (i, j = 1, 2…n)
Damage degree of the accident (fire) A	fire-causative factors B1	0.558	human behavior C11	0.649	0.362
			combustibles C21	0.279	0.156
			open fire C31	0.072	0.040
	equipment operation factors B2	0.122	equipment load C12	0.316	0.039
			equipment condition C22	0.342	0.042
			heat C32	0.146	0.018
			ventilation C42	0.134	0.016
			maintenance C52	0.062	0.008
	firefighting factors B3	0.320	rescue speed C13	0.369	0.118
			staff assignment C23	0.369	0.118
			fire equipment C33	0.174	0.056
			fire alarm C43	0.088	0.028

## References

[1] Karlsson B., Quintiere J.G. (2000). Enclosure Fire Dynamics.

[2] Comprehensive News: The Fire in Russia’s Shopping Center Has Killed 64 People, and Relevant Departments Have Stepped up Investigation to Eliminate Hidden Dangers. http://www.xinhuanet.com/world/2018-03/27/c_1122594262.htm.

[3] A Fire Broke out in a Shopping Mall in Qingyuan, Guangdong. The Whole Building Was Engulfed by the Fire. http://news.china.com.cn/live/2020-08/18/content_928342.htm.

[4] Fong N.K., Wong K.C. (1988). Statistical Data for Fires in Hong Kong and Preliminary Views on Building Fire Risk Analysis. Fire Saf. Sci..

[5] Brannigan V., Meeks C. (1995). Computerized Fire Risk Assessment Models: A Regulatory Effectiveness Analysis. J. Fire Sci..

[6] Zhou W. (1997). Fire Hazard Assessment in a Big Hall with the Multi-Cell Zone Modelling Concept. J. Fire Sci..

[7] Yao W., Huang H., Shen S., Qiao L., Wang W., Zhang H. (2013). Fire risk mapping based assessment method applied in performance based design. Fire Saf. J..

[8] Che H., Ding W.P., Cheng J. Assessment for the fire risk of the underground parking area. Proceedings of the International Conference on Manufacturing and Engineering Technology.

[9] Liu F., Zhao S., Weng M., Liu Y. (2017). Fire Risk Assessment for Large-scale Commercial Buildings Based on Structure Entropy Weight Method. Saf. Sci..

[10] Yang J., Chen Y. (2014). Research and Application of Fire Risk Assessment System for Marketplace Buildings. Proc. Eng..

[11] Lau C.K., Lai K.K., Lee Y.P., Du J. (2015). Fire risk assessment with scoring system, using the support vector machine approach. Fire Saf. J..

[12] Mearns A.B. Fault Tree Analysis: The study of unlikely events in complex systems. Proceedings of the Boeing/UW System Safety Symposium.

[13] Haasl D.F. Advanced Concepts in Fault Tree Analysis. Proceedings of the Boeing/UW System Safety Symposium.

[14] IEC (2006). IEC 61025:2006, Fault Tree Analysis.

[15] Kabir S. (2017). An overview of fault tree analysis and its application in model based dependability analysis. Expert Syst. Appl..

[16] Jung S.J., Yoo J., Lee Y.J. (2020). A Software Fault Tree Analysis Technique for Formal Requirement Specifications of Nuclear Reactor Protection Systems. Reliab. Eng. Syst. Saf..

[17] Hu Y.N. (2016). Research on the Application of Fault Tree Analysis for Building Fire Safety of Hotels. Proc. Eng..

[18] Saaty T. (1980). The Analytic Hierarchy Process.

[19] Tang F., Hu L., Huo R., Xu Y., Zhu S., Yao B. (2010). Urban village regional fire risk assessment model based on AHP. Fire Sci. Technol..

[20] Tian Y., Cai J. (2009). Study on application of AHP in fire risk evaluation of marketplace. J. Catastrophol..

[21] Xu Z., Liu D., Cao H., Fu R. (2015). Study on fire risk assessment of historic buildings based on AHP. J. Rail. Sci. Eng..

[22] Jia X., Gao Y., Wei B., Wang S., Tang G., Zhao Z. (2019). Risk Assessment and Regionalization of Fire Disaster Based on Analytic Hierarchy Process and MODIS Data: A Case Study of Inner Mongolia, China. Sustainability.

[23] Notice on Determining High Risk Fire Units and Key Fire Safety Units in Qingshan District (by Qingshan District Fire Rescue Brigade). http://www.qingshan.gov.cn/xxgk/gsgg/202005/t20200515_1320965_app.shtml.

